# Cross-Alloy Transferability of Spindle-Synchronous Vibration Signatures in CNC Milling Using Physics-Informed Autospectrum Features

**DOI:** 10.3390/ma19204341

**Published:** 2026-10-15

**Authors:** Muhammed İşci

**Affiliations:** Mechatronics Program, Department of Machine and Metal Technologies, Technical Sciences Vocational School, Kayseri University, Kayseri 38280, Turkey; muhammedisci@kayseri.edu.tr

**Keywords:** Al 6061/7075, CNC milling, vibration response, autospectrum, spindle speed, explainable ML

## Abstract

Data-driven recognition of machining conditions in CNC milling processes represents a significant research focus within process monitoring applications. This work assesses whether physics-informed, spindle-synchronous vibration representations remain transferable between Al 6061 and Al 7075 using multi-axis and multi-location autospectrum data. Spindle speed is used as a controlled and physically traceable process-state label rather than as an unknown CNC variable to be reconstructed. Vibration signals were collected under various combinations of feed rate, spindle speed, and depth of cut. Subsequently, autospectrum descriptors associated with spindle rotation, harmonic components, frequency-band energy, and sensor location were extracted. Preliminary cross-alloy analysis demonstrated that spindle speed recognition exhibited more stable transfer performance than feed rate or depth of cut recognition. Multiple machine learning models were evaluated, with the combination of spindle-related autospectrum descriptors + LightGBM model selected as the final configuration. The resulting model achieved 0.8708 accuracy, 0.8701 Macro-F1, 0.7500 MCC, and 0.9615 ROC-AUC in the cross-alloy spindle speed recognition task. In two-way transfer analyses, Macro-F1 scores of 0.8748 and 0.8661 were obtained for the Al 6061 to Al 7075 and Al 7075 to Al 6061 scenarios, respectively. Ablation studies indicated that spindle-related frequency descriptors offer advantages over comprehensive feature representations. SHAP-based explainability analysis revealed that harmonic band, tooth-passing band, and sensor-location-dependent spectral features were most influential in model decisions. These findings indicate that physics-informed autospectrum descriptors preserve discriminative spindle-synchronous vibration information across the investigated Al 6061 and Al 7075 alloy domains.

## 1. Introduction

CNC milling is a widely utilized machining method in contemporary manufacturing, offering precise and adaptable processing of diverse engineering materials. The dynamic interaction between the cutting tool and the workpiece during milling generates complex vibration responses that indicate the instantaneous state of the machining process. These vibrations are intrinsically linked to cutting stability, tool-workpiece interaction, surface quality, tool condition, and overall process reliability. Consequently, monitoring vibrations induced by milling yields critical information for understanding machining behavior and for the development of data-driven process monitoring systems. Accelerometer-based vibration measurements are a prevalent and effective technique for capturing dynamic responses under varying cutting conditions and for assessing changes in process parameters. Wszołek et al. reported that spontaneously induced vibrations in CNC machines are linked to precision errors, poor surface quality, tool wear, and potential machine damage [1]. Zhang et al. found that vibration signals in the milling process serve as effective monitoring data for reflecting tool status and changes in machining conditions [2]. Lai et al. observed that surface roughness during milling is influenced by cutting parameters and vibrations generated during machining, indicating that vibration information should be incorporated into surface quality monitoring models [3]. Cardoso et al. highlighted the significance of RMS- and FFT-based analyses of vibration signals in real-time process monitoring and optimization studies [4]. Chuangwen et al. demonstrated that parameters such as spindle speed, feed rate, and cutting depth affect milling vibrations [5]. These findings suggest that vibration responses generated during milling can be utilized not only for process monitoring but also for identifying machining conditions such as cutting parameters and spindle speed. Bediaga et al. established that the frequency response obtained during milling is directly related to tool rotation frequency and spindle speed, making spindle speed selection a critical variable for process stability [6]. Chu et al. showed that machining conditions can be identified with high accuracy using ApEn and STFT-based features extracted from sound and vibration signals in CNC milling [7]. Wang et al. demonstrated that vibration signals contain information about cutting parameters such as spindle speed, feed rate, and cutting depth in the milling process, and these signals can be used for cutting condition classification [8]. Pasandidehpoor et al. noted that sensor data, including vibration, cutting force, acoustic emission, and motor current, are widely used in machine learning-based monitoring systems for CNC milling processes [9]. The identification of cutting parameters and spindle speed from vibration signals requires that these signals be represented in both the time and frequency domains. Frequency-domain, spectral, and autospectrum-based representations are therefore essential for characterizing process behavior in milling vibration analyses. Chandan et al. demonstrated that FFT-based frequency-domain features effectively support CNC tool state classification by capturing the energy distribution and spectral structure in vibration signals [10]. Shoaib et al. found that temporal and spectral features derived from vibration and acoustic emission signals in CNC milling provide effective representations for tool wear prediction [11]. Alberts et al. reported that FFT-based vibration features are suitable for machining chatter classification using the Random Forest model [12]. Liu et al. showed that vibration energy in milling operations can be monitored via the power spectrum, and that changes in energy distribution can be used to classify stable, transition, and chatter states [13]. Ozkirimli and Ozturk highlighted the importance of frequency domain measurements in machining stability analysis by employing cross-power spectrum and force signal autospectrum representations in milling tool FRF calculations [14]. Olejárová et al. observed that milling vibrations are related to spindle speed, and that frequency spectrum analysis can reveal process-induced characteristic vibration components [15]. Hortobágyi et al. found that spindle speed variation in CNC milling significantly affects the amplitude spectrum, and that FFT-based vibration analysis can be used to monitor process parameters [16]. Although frequency domain representations reveal the spectral structure in milling vibration signals, these representations must be converted into features associated with physical processes, such as spindle rotation, harmonics and sensor location, to be more meaningful in modeling. Mohamadyari Heydarlou et al. argued that physically significant features, such as milling force coefficients, can transform raw signals dependent on process conditions into more generalizable and interpretable feature representations [17]. Wu et al. demonstrated that machine learning models informed by physical information for milling operations can reduce data dependency and yield results more consistent with physical laws [18]. Huang et al. showed that integrating milling dynamics information into a machine learning model can improve chatter prediction performance compared to purely data-driven models [19]. Beyond frequency domain and physics-informed feature extraction, the location of sensors collecting vibration data is a critical factor that directly influences machining monitoring performance. Tran et al. demonstrated that combining vibration and sound signals for milling chatter detection yields more comprehensive process information and enhances classification performance compared to single-sensor approaches [20]. Li et al. found that spindle-generated frequency components in CNC grinding systems are transmitted to various measurement points through the machine bed and axis structures, indicating that both sensor placement and transmission path significantly affect the monitored frequency content [21]. When vibration features, which are linked to physical processes and collected from multiple sensors, are input into machine learning models for machining condition classification, this facilitates automatic and data-driven identification of cutting conditions. Wang et al. established that milling conditions can be classified using vibration-based features and machine learning techniques, and that variations in spindle speed have a pronounced effect on these features [22]. Çekik and Turan demonstrated that vibration data from CNC milling can be effectively utilized in deep learning-based classification models for process monitoring and anomaly detection [23]. Çetinkaya et al. showed that artificial neural network models can accurately predict machining vibration behavior using data measured under varying material, spindle speed, and feed rate conditions in CNC turning processes [24]. However, the reliability of machining condition classification models extends beyond performance under identical experimental conditions; maintaining discriminative capability across different material types, process conditions, and data distributions is essential for cross-alloy generalization, transferability, and domain adaptation. Alsoufi and Bawazeer compared machine learning model performance across various engineering alloys, demonstrating that model effectiveness in machining processes depends on material properties and that cross-alloy evaluation protocols are vital for model selection [25]. Ebrahimi Araghizad et al. found that incorporating thermomechanical properties into material definitions can enhance the transferability of machine learning models to novel materials in milling processes [26]. In machining monitoring research, reliability and engineering interpretability are as important as model generalizability under diverse material and process conditions, as well as the explainability of the vibration features that inform model decisions. Xue et al. quantitatively assessed the contribution of input features to model outputs using SHAP analysis in milling tool wear prediction, demonstrating that this method improves the interpretability of deep learning-based machining monitoring models [27]. Li et al. developed a physics-informed, interpretable machine learning approach for depth of cut (DOC) monitoring in peripheral milling, showing that a model structure based on physically meaningful dimensionless force features can deliver reliable monitoring performance even with limited training data [28]. Recent research highlights the growing significance of vibration-based sensing, frequency-domain representation, physics-informed feature extraction, multi-sensor monitoring, machine learning classification, cross-alloy generalization, and explainable AI in machining monitoring. Despite these advancements, limited attention has been given to whether physically anchored vibration signatures learned under one workpiece alloy remain discriminative when the material domain changes. In this study, spindle speed is not treated as an unknown CNC variable to be reconstructed. Instead, the two commanded spindle speeds are used as controlled and physically traceable process states. Classification between these states is employed as a quantitative probe of the cross-alloy transferability of spindle-synchronous vibration information. Accordingly, the primary objective is to determine whether physics-informed autospectrum representations preserve process-state discrimination when the workpiece alloy changes between Al 6061 and Al 7075.

## 2. Materials and Methods

This section outlines the analysis of vibrations generated during the CNC milling process within the context of cross-alloy spindle speed recognition. Localized vibration monitoring, particularly in the spindle, tool holder, and bed regions, is essential for ensuring machining stability and production efficiency in CNC milling operations. In this study, multi-channel vibration data collected during the machining of Al 6061 and Al 7075 alloys were evaluated using autospectrum representations derived from the PULSE environment. The methodology includes defining the experimental setup and cutting conditions, extracting physically meaningful spectral features from autospectrum data, establishing a cross-alloy validation protocol, and comparing multiple machine learning models. Additionally, analyses of frequency bands, sensor configurations, top-k feature reduction, feature importance, and SHAP values were performed to assess the robustness and interpretability of the final model.

### 2.1. Experimental Setup and Workpiece Materials

Experimental studies utilized a three-axis CNC milling machine capable of machining along the x, y, and z axes. Vibrations generated during the milling process were monitored using a Brüel & Kjær 4524-B-001 (Brüel & Kjær, Nærum, Denmark) tri-axial accelerometer mounted in the tool holder or spindle area. Additional accelerometers were employed to capture vibration data from the machine body and bed. The collected vibration data were transferred to a computer via a Brüel & Kjær 3560-B (Brüel & Kjær, Nærum, Denmark) Intelligent Data Acquisition system and subsequently processed using PULSE software (LabShop Version 11.1). The technical specifications of the CNC milling machine are provided in Table 1.

**Figure 1:**
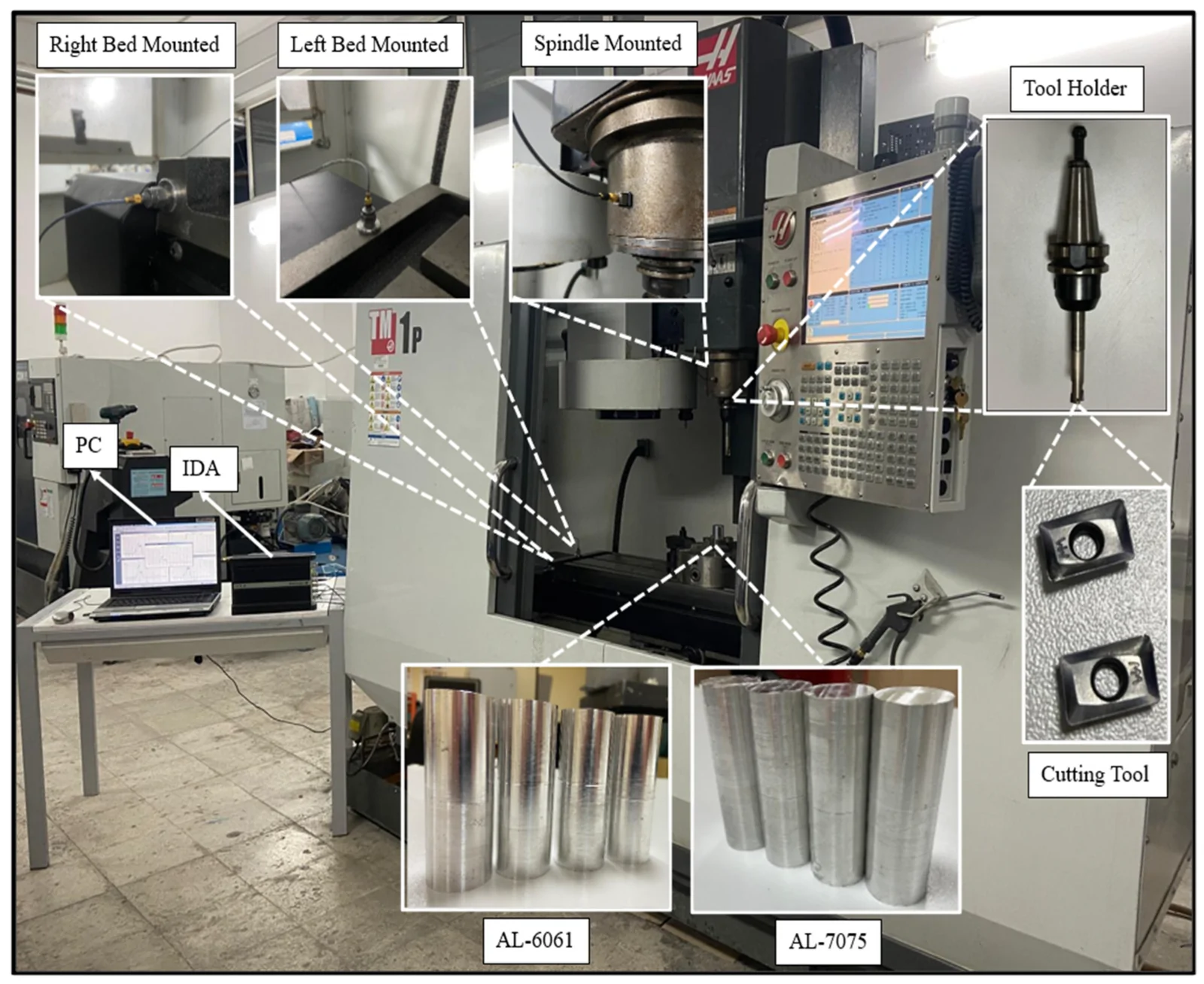
Experimental setup, sensor placement, workpiece materials, and cutting tool configuration.

The experimental setup, sensor placement, workpiece specimens, and tool holder-insert configuration are shown in Figure 1. The milling experiments were performed using a 16 mm diameter indexable end mill equipped with two active APKT 100304 PDTR carbide inserts. The cutter had an overall length of 100 mm, a cutting-section length of 25 mm, and a maximum axial cutting capacity of 9 mm. Each insert had a nominal corner radius of 0.40 mm. The same cutter and insert configuration was maintained throughout all experiments.

Two different aluminum alloys widely used in manufacturing applications—Al 6061 and Al 7075—were employed as workpiece materials. The cutting process was performed by mounting the milling cutter insert onto the tool holder. All experiments were conducted under dry cutting conditions to eliminate any potential effects of coolant on material behavior and vibration characteristics.

### 2.2. Cutting Conditions

Milling experiments were conducted using various combinations of spindle speed, feed rate, and depth of cut parameters. The experimental design incorporated two levels each for spindle speed, feed rate, and depth of cut, resulting in a total of eight distinct cutting conditions. These eight conditions were applied separately to Al 6061 and Al 7075 materials, yielding a total of 16 experimental data records. As the study’s ultimate classification objective was spindle speed recognition, the spindle speed value served as the primary target variable, while feed rate and depth of cut data were recorded to define the experimental conditions and for use in preliminary target screening analyses. The cutting conditions established through the combinations of spindle speed, feed rate, and depth of cut parameters used in the experiments are presented in Table 2.

Based on the nominal cutter diameter of 16 mm, the corresponding cutting speeds were 75.4 m/min at 1500 rpm and 100.5 m/min at 2000 rpm. Thanks to this experimental design, spindle speed classification was evaluated under various feed rate and depth of cut levels, and the model’s cross-alloy generalization performance was examined without being limited to a specific cutting condition.

### 2.3. Vibration Data Acquisition and Autospectrum Representation

Vibration data generated during milling operations were collected through multiple channels located at the tool holder/spindle region and the machine bearing region. The measurement system recorded vibration components along the X, Y, and Z axes from the tool holder region, and captured left and right bearing vibration responses from the bearing region. Consequently, five distinct vibration channels were evaluated in each experiment. The vibration data were analyzed using processed autospectrum frames exported from the Brüel & Kjær PULSE environment. Each autospectrum frame was considered an observation that represented the frequency-domain response of the five vibration channels within the corresponding time interval. Table 3 provides basic information regarding the autospectrum data structure employed.

**Figure 2:**
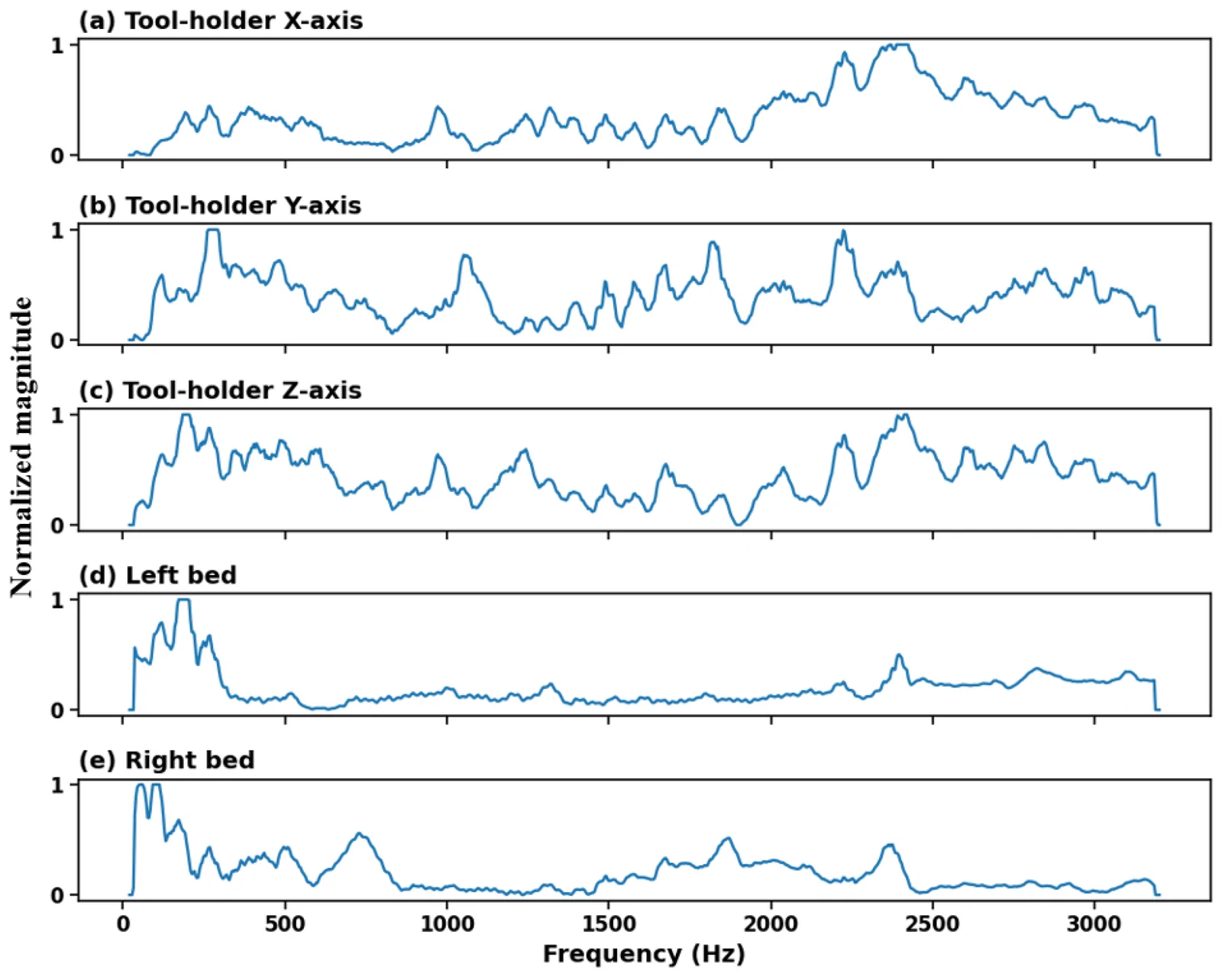
Representative normalized autospectrum responses obtained from the vibration channels. (**a**) Tool-holder *X*-axis, (**b**) tool-holder *Y*-axis, (**c**) tool-holder *Z*-axis, (**d**) left bed, and (**e**) right bed.

Table 3 provides an overview of the autospectrum data structure utilized in this study. The dataset comprises 39,948 frame-level autospectrum observations collected from 16 experimental files. Each observation contains 801 frequency components at a resolution of 4 Hz, spanning the 0 to 3.2 kHz frequency range. Considering all five vibration channels, the analysis includes a total of 159,991,740 autospectrum values. Figure 2 displays representative autospectrum responses from the five vibration channels, illustrating frequency-domain responses from the X, Y, and Z axes in the tool holder and spindle region, as well as from the left and right bearing regions.

**Figure 3:**
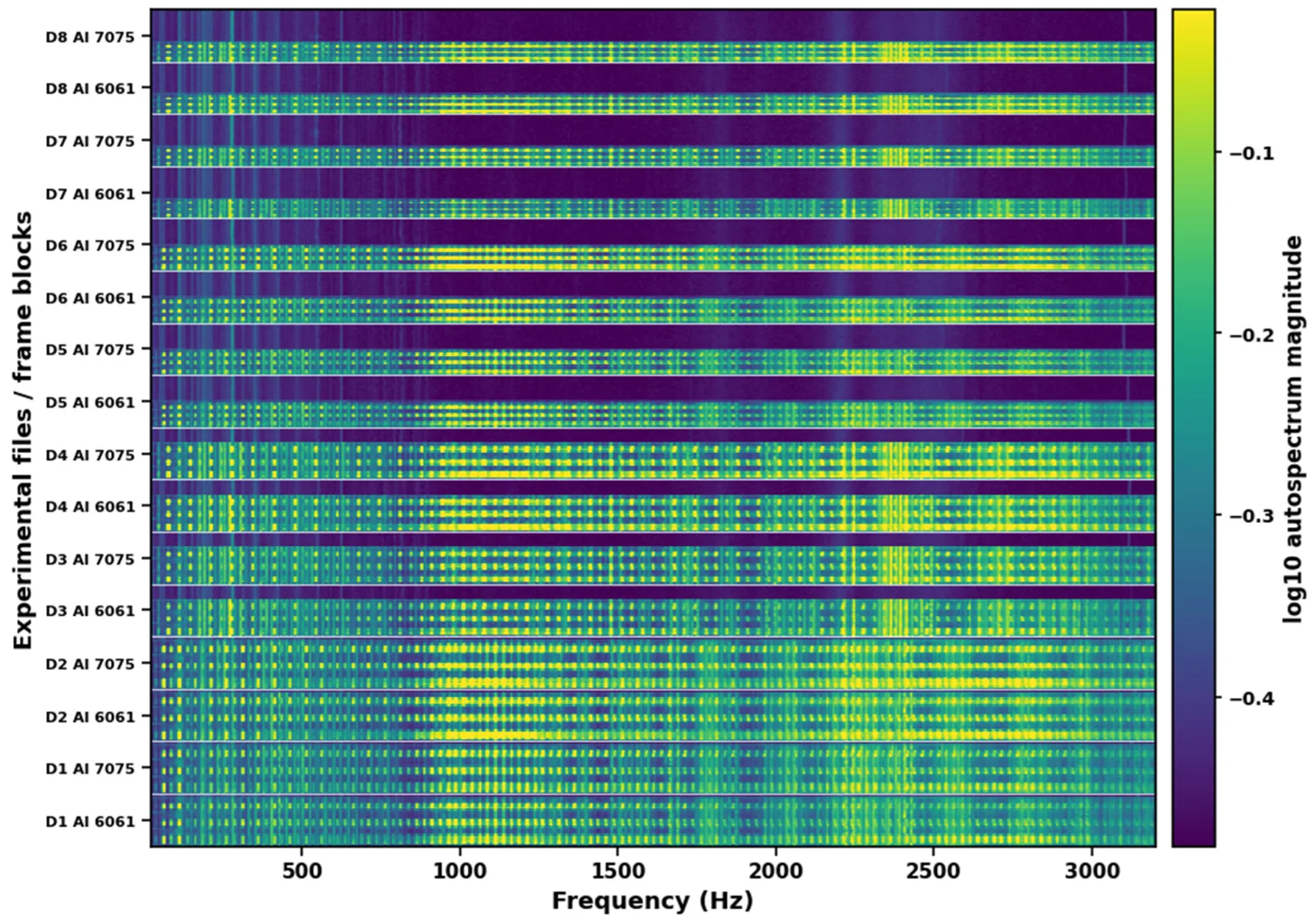
Overall autospectrum heatmap across all experimental files and frame-level observations.

Figure 2 demonstrates that sensor locations yield distinct autospectrum characteristics in the frequency domain. Variations across a broader frequency range are evident at the tool-holder axes, whereas sensors positioned in the bearing region display more limited but still distinctive spectral responses within specific frequency bands. These findings support the use of multi-channel analysis for fundamental frequency components related to spindle speed, harmonic regions, and tooth-passing frequency bands. For improved visualization, the representative autospectrum responses in Figure 2 were normalized and smoothed for each channel. Feature extraction and machine learning analyses, however, utilized the raw autospectrum values exported from the PULSE environment. The overall frequency-domain distribution of all frame-level autospectrum observations is shown as a heatmap in Figure 3. To enhance readability, frame-level observations within each experimental file were visualized using a block-averaging method.

Figure 3 presents frequency on the horizontal axis and experimental data-based frame blocks for Al 6061 and Al 7075 materials under experimental conditions D1–D8 on the vertical axis. The color scale indicates the magnitude of the logarithmic autospectrum. The visualization demonstrates that spectral energy distribution differs across experimental conditions and material types, indicating a broad frequency-domain structure within the dataset. Block averaging was implemented exclusively for visualization; feature extraction, classification, and explainability analyses utilized the raw autospectrum values exported from the PULSE environment.

### 2.4. Data Organization and Labeling

The autospectrum data were organized according to experiment file, material type, cutting condition, and vibration channel information. Each experiment file represents a unique combination of material and cutting condition. Cutting conditions D1 through D8 were evaluated separately for Al 6061 and Al 7075, resulting in a total of 16 experimental files included in the analysis. Each autospectrum frame is labeled with the corresponding experiment file information. Accordingly, material type, condition identification, feed rate, spindle speed, and depth of cut are assigned to each frame. The primary classification objective is spindle speed recognition, with spindle speed used as a binary target variable (1500 rpm and 2000 rpm). Feed rate and depth of cut data are retained to define experimental conditions and for preliminary target screening analyses. The labeling structure used in the modeling process, including value ranges and the role of each label field, is presented in Table 4.

This labeling structure ensures that each frame-level autospectrum observation is linked to both the material type and the corresponding experimental cutting condition. The frame-level observations were not considered independent machining experiments; rather, each frame was treated as a frequency-domain observation derived from its respective experiment file. As a result, both the classification objective and the cross-alloy evaluation framework were explicitly defined during the modeling process. This approach allowed the classification task to be analyzed within a controlled cross-alloy evaluation framework while maintaining the association between each frame-level observation and its specific experimental condition.

### 2.5. Feature Extraction from Autospectrum Data

**Figure 4:**
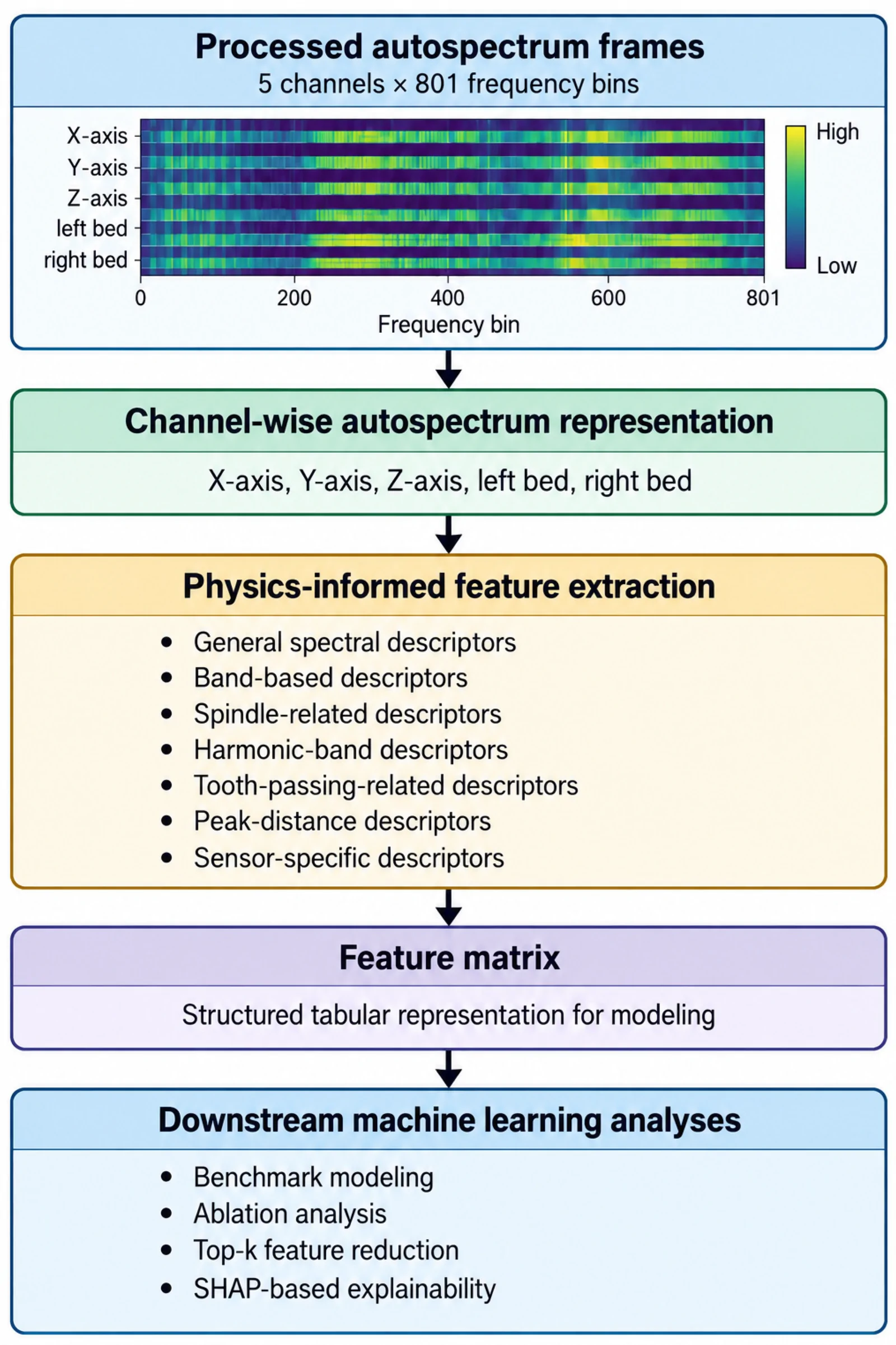
Physics-informed feature extraction pipeline from autospectrum representations.

Frequency-domain features are extracted from autospectrum frames for application in machine learning models. Each frame is considered a multi-channel autospectrum representation, comprising 801 frequency components across five vibration channels. The feature extraction process aims to capture both the overall spectral distribution and frequency regions that are physically associated with spindle speed. Figure 4 schematically illustrates the extraction of physically meaningful features from autospectrum frames and their integration into the modeling process.

Figure 4 presents the workflow from channel-wise organization of processed autospectrum frames through physically informed feature extraction to their integration into machine learning analyses. Initially, autospectrum representations for the X, Y, and Z axes, as well as the left and right bearing channels, were evaluated separately. Multiple feature groups were then extracted, including general spectral descriptors, band-based descriptors, spindle-related descriptors, harmonic-band descriptors, tooth-passing-related descriptors, peak-distance descriptors, and sensor-specific descriptors. General spectral descriptors summarize the overall frequency-domain distribution for each channel. Band-based descriptors quantify total, maximum, and ratio-based spectral magnitudes within selected frequency bands. Spindle-related descriptors capture variations in frequency regions associated with spindle speed. Harmonic-band and tooth-passing-related descriptors represent periodic vibration components linked to rotational motion and cutting-edge passes. Peak-distance descriptors measure the distance of dominant spectral peaks from predefined frequency regions, while sensor-specific descriptors retain the unique contribution of each sensor location. This feature extraction process transforms each frame-level autospectrum observation into a structured feature vector. The resulting feature matrix is then used in machine learning analyses, serving as a standardized input for benchmark model comparisons, frequency-band ablation, sensor ablation, top-k feature reduction, and SHAP-based explainability analyses.

### 2.6. Preliminary Target Screening

A preliminary target screening analysis was conducted prior to the modeling process to determine the final classification task. Three process parameters—spindle speed, feed rate, and depth of cut—were evaluated as potential classification targets. The aim was to identify the target variable with the greatest stability and highest cross-alloy transferability between the Al 6061 and Al 7075 material domains. Each target variable was assessed using a consistent autospectrum-based feature extraction method and cross-alloy evaluation protocol. This approach ensured that observed performance differences were attributable to the discriminative capacity of the autospectrum representations for each process parameter, rather than to data partitioning or evaluation inconsistencies. The preliminary assessment indicated that spindle speed classification demonstrated more stable and robust cross-alloy performance than feed rate or depth of cut. Therefore, cross-alloy spindle speed recognition was selected as the primary classification objective for this study. Feed rate and depth of cut were retained as supplementary, exploratory screening tasks. The results of the preliminary target screening are presented in the “Results” section; this analysis was conducted exclusively to justify the selection of spindle speed as the final classification target.

### 2.7. Cross-Alloy Validation Protocol

A cross-alloy validation protocol was implemented to evaluate the model’s generalization capability across distinct material domains. In this protocol, training and testing datasets originated from different materials; specifically, the model was trained on one material and evaluated on another. This methodology assessed both the model’s performance within a single data distribution and its transferability to alternative material domains. A bidirectional validation scenario was established: in the first case, the model was trained on Al 6061 data and tested on Al 7075 data; in the second, the training and testing materials were reversed. This design enabled assessment of whether model performance was influenced by the direction of material transfer. To ensure the target material remained an entirely unseen test domain, all target material data were excluded from the training process. This strategy was adopted to avoid the overly optimistic performance estimates that can result from random frame-level data splitting and to provide a more rigorous evaluation of generalization. To prevent information leakage arising from correlated frames within the same machining experiment, the source-domain training and validation subsets were separated at the experimental-file level rather than by random frame-level splitting. In the implemented protocol, conditions D1 and D3 of the source alloy were reserved entirely for validation, while all remaining source-alloy experimental files were used for training. Because each alloy–condition combination corresponds to a unique experimental file, this condition-based partitioning is equivalent to experimental-file-level grouping. Therefore, all frames originating from a given experimental file were assigned exclusively to either the training or validation subset. In addition, the complete target-alloy dataset was kept entirely unseen during training, scaling, threshold selection, and model selection. Feature scaling parameters were estimated exclusively from the source-domain training data and subsequently applied unchanged to the source-validation and target-test datasets. Because frames originating from the same experimental file may exhibit within-experiment correlation, they were not interpreted as independent machining trials. Entire experimental files were kept intact during data partitioning, ensuring that correlated frames from a given trial were never distributed across training, validation, or test subsets. The resulting bidirectional performance metrics were analyzed with respect to average performance, class-specific error distribution, and consistency across transfer directions. Final model selection prioritized balanced performance across both cross-alloy scenarios rather than success in a single transfer direction.

### 2.8. Machine Learning Models

A range of machine learning models was evaluated to compare classification approaches for cross-alloy spindle speed recognition. The selected models represent linear decision boundaries, kernel-based classification, artificial neural networks, and tree-based ensemble or boosting algorithms. This evaluation aimed to determine whether model performance is influenced by the choice of algorithm family. Each frame-level autospectrum observation, obtained through feature extraction, is represented as a structured feature vector in Equation (1):

*x_i_* = [*x_i_*_1_, *x_i_*_2_, …,*x_ip_*] (1)

Here, *x_i_* represents the *p*-dimensional feature vector corresponding to the *i*. frame-level autospectrum observation. Since the main target variable of the study is spindle speed, the class label is defined in a binary manner, as shown in Equation (2):

*y_i_* ∈ {1500 rpm, 2000 rpm} (2)

In Equation (3), the classification models are treated as a decision function that maps the input feature vector to the spindle speed class.

(3) $${\hat{y}}_{i}=f_{m}\left(x_{i}\right)$$

Here, *f_m_* represents the machine learning model being evaluated, while ${\hat{y}}_{i}$ denotes the predicted spindle speed class. In probability-based models, the class prediction is made based on the prediction probability associated with the respective class, as shown in Equation (4):

(4) $${\hat{y}}_{i}=\{\begin{array}{c} 2000\;\mathrm{rpm},\;P(y_{i}=2000|x_{i})\geq \tau \\ 1500\;\mathrm{rpm},\;P(y_{i}=2000|x_{i})<\tau \end{array}$$

Here, *τ* represents the decision threshold. The machine learning models evaluated in the study are presented in Table 5.

All models were evaluated using the same autospectrum-based feature matrix and cross-alloy validation protocol. To ensure reproducibility, a fixed random seed *SEED* = 42 was used for all applicable models. Prior to model training, features were scaled using StandardScaler; the scaler was fitted solely on the training data and subsequently applied to the source-validation and target-test datasets. The decision threshold was selected based exclusively on the validation data from the source material; the target material was not utilized during the training, validation, or threshold tuning stages. The key hyperparameter settings employed are summarized in Table 6.

The hyperparameters listed in Table 6 serve as the baseline model settings, which remain constant throughout the benchmark, frequency-band ablation, sensor ablation, and top-k feature reduction analyses. This methodology ensures that model comparisons are based exclusively on differences in algorithm family and feature representation. The model benchmarking process is depicted in Algorithm 1.
**Algorithm 1:** Machine learning benchmark and final model selection for cross-alloy spindle speed recognition*Input:* Frame-level autospectrum descriptors, spindle speed labels, alloy labels, and candidate machine learning models *Output:* Final model configuration for cross-alloy spindle speed recognition *Begin* *Prepare* the autospectrum-based feature matrix from frame-level frequency-domain descriptors. *Define* the bidirectional cross-alloy transfer scenarios:    Al 6061 → Al 7075    Al 7075 → Al 6061 *For each* transfer scenario *do*    *Assign* one alloy as the source domain.    *Assign* the other alloy as the unseen target domain.    *Split the source-domain data at the experimental-file level, reserving complete source experimental files for validation.*    *Standardize* the descriptors using only the source-domain training data.    *For each* candidate machine learning model *do*    *Train* the model using the source-domain training data.    *Determine* the decision threshold using the source-domain validation data.    *For each* target-alloy test observation *do*       *If* predicted probability ≥ decision threshold *then*       *Assign* the observation to **2000 rpm**.       *Else*       *Assign* the observation to **1500 rpm**.       *End if*    *End for*    *Compute* Accuracy, Balanced Accuracy, Macro-F1, MCC, ROC-AUC, and PR-AUC.    *End for* *End for* *Compare* the bidirectional transfer performance of all candidate models. *Perform* frequency-band, sensor, and top-k feature ablation analyses. *If* a configuration provides balanced transfer performance, compactness, and physical interpretability *then*  *Select* it as the final model configuration. *End if* *Return* **spindle-related autospectrum descriptors + LightGBM**. *End*

*Note.* The same validation protocol is applied to all candidate models to ensure a consistent comparison across model families.

Algorithm 1 details the process, beginning with the preparation of an autospectrum-based feature matrix, for training, testing, and comparing candidate machine learning models using a two-way cross-alloy validation protocol. This framework systematically delineates the steps of training, validation, scaling, decision threshold determination, testing on the target alloy, and performance metric calculation. The protocol ensures that model comparisons are performed consistently and that the final configuration is selected based on performance values as well as criteria such as transfer consistency, model compactness, and physical interpretability. Model performance was assessed using true positive (*TP*), true negative (*TN*), false positive (*FP*), and false negative (*FN*) values derived from the confusion matrix. For the binary spindle speed recognition task, the primary performance metrics were calculated as presented in Equations (5)–(8) below:

(5) $$\mathrm{Accuracy}=\frac{\mathrm{TP}+\mathrm{TN}}{\mathrm{TP}+\mathrm{TN}+\mathrm{FP}+\mathrm{FN}}$$

(6) $$\mathrm{Precision}=\frac{\mathrm{TP}}{\mathrm{TP}+\mathrm{FP}}$$

(7) $$\mathrm{Recall}=\frac{\mathrm{TP}}{\mathrm{TP}+\mathrm{FN}}$$

(8) $$F1=\frac{2\times \mathrm{Precision}\times \mathrm{Recall}}{\mathrm{Precision}+\mathrm{Recall}}$$

In binary classification, the macro-F1 value is obtained as the average of the F1 scores calculated for both classes, as shown in Equation (9):

(9) $$\mathrm{Macro}\text{-}F1=\frac{1}{C}\sum_{C=1}^{C}{F1}_{C}$$

Balanced accuracy, on the other hand, is calculated as the average of class-based sensitivity values, as shown in Equation (10):

(10) $$\mathrm{Balanced}\;\mathrm{Accuracy}=\frac{1}{2}\left(\frac{\mathrm{TP}}{\mathrm{TP}+\mathrm{FN}}+\frac{\mathrm{TN}}{\mathrm{TN}+\mathrm{FP}}\right)$$

To more robustly evaluate the model’s error balance, the Matthews correlation coefficient (*MCC*) given in Equation (11) was used:

(11) $$\mathrm{MCC}=\frac{\mathrm{TP}\times \mathrm{TN}-\mathrm{FP}\times \mathrm{FN}}{\sqrt{\left(\mathrm{TP}+\mathrm{FP})(\mathrm{TP}+\mathrm{FN})(\mathrm{TN}+\mathrm{FP})(\mathrm{TN}+\mathrm{FN}\right)}}$$

Cohen’s kappa was used to assess the difference between observed accuracy and chance agreement, as shown in Equation (12):

(12) $$\kappa =\frac{p_{o}-p_{e}}{1-p_{e}}$$

Here, *p_o_* represents the observed agreement, while *p_e_* represents the expected chance agreement. For probability-based evaluation, the log-loss and Brier score metrics given in Equations (13) and (14) were used:

(13) $$\mathrm{Log}\text{-}\mathrm{loss}=-\frac{1}{N}\sum_{i=1}^{N}\left[y_{i}\log\left({\hat{p}}_{i}\right)+\left(1-y_{i}\right)\log\left(1-{\hat{p}}_{i}\right)\right]$$

(14) $$\mathrm{Brier}\;\mathrm{score}=\frac{1}{N}\sum_{i=1}^{N}{\left({\hat{p}}_{i}-y_{i}\right)}^{2}$$

Here, N represents the total number of observations; *y_i_* denotes the true class label; and ${\hat{p}}_{i}$ represents the predicted probability for the corresponding class. The ROC-AUC and PR-AUC values were calculated as the areas under the ROC and precision-recall curves, respectively. The spindle-speed recognition task was binary, with 1500 rpm and 2000 rpm as the two classes. Accordingly, ROC-AUC was calculated using the true binary labels and the predicted probability of the 2000 rpm class across all possible decision thresholds; no multi-class averaging strategy was applied. Since ROC-AUC is threshold-independent, its value can be higher than threshold-dependent metrics such as Macro-F1 and balanced accuracy. During the benchmarking phase, all models were compared using a comprehensive feature set. Model performance was subsequently evaluated across different feature representations, including analyses of frequency bands, sensor configurations, and top-k feature reduction. LightGBM was selected as the final model not by prior designation, but because it demonstrated the most balanced performance, particularly when spindle-related autospectrum descriptors were employed, as determined by benchmark and ablation analyses. This methodology ensured that model selection was informed by both physically meaningful feature representations and robust cross-alloy generalization, rather than by algorithmic preference alone.

### 2.9. Frequency-Band, Sensor, and Top-k Feature Ablation Analyses

Three distinct ablation analyses were conducted: frequency-band ablation, sensor ablation, and top-k feature reduction. These analyses aimed to determine whether the model’s performance depended exclusively on the combined use of all features or if comparable results could be achieved with more compact, physically meaningful, or sensor-reduced representations.

#### 2.9.1. Frequency-Band Ablation

The frequency-band ablation analysis involved evaluating distinct groups of frequency-domain features derived from autospectrum data individually. Representation groups based on physically meaningful frequency regions, including spindle-related bands, and harmonic frequency regions, were compared to an all-feature representation. This approach aimed to determine which frequency regions provided the most discriminative information for spindle speed recognition. The spindle-related frequency regions were not introduced to infer an unknown spindle-speed command. Instead, the known commanded spindle speeds provided physically anchored reference frequencies for assessing whether spindle-synchronous vibration information remained transferable across alloy domains. Accordingly, rotational, tooth-passing, and harmonic components were treated as physical markers of the controlled process states, while cross-alloy classification performance was used to quantify the preservation of their discriminative information after the material domain changed. For spindle speeds of 1500 rpm and 2000 rpm, the fundamental rotational frequencies were calculated as follows:

(15) $$f_{r}=\frac{n}{60}$$

Here, *f_r_* represents the spindle rotational frequency, while n denotes the spindle speed in rpm. Accordingly, the fundamental rotational frequency was determined to be 25 Hz for 1500 rpm and approximately 33.33 Hz for 2000 rpm. The physics-informed frequency regions were predefined from spindle kinematics and the two-insert cutter configuration. The rotational frequencies were 25 and 33.33 Hz, yielding TPF values of 50 and 66.67 Hz and third-order harmonics of 75 and 100 Hz for 1500 and 2000 rpm, respectively. Considering the 4 Hz autospectrum resolution, fixed neighboring bands were defined around these components and were not optimized using target-alloy test data. Harmonic-band descriptors were extracted from frequency regions surrounding the harmonics of these fundamental frequencies. Thus, the feature extraction process was based not only on the general spectral distribution but also on frequency components physically related to the spindle speed.

#### 2.9.2. Sensor Ablation

The sensor ablation analysis evaluated the impact of different sensor configurations on classification performance. A configuration utilizing all sensors was compared with setups involving sensors located in the tool holder or spindle region, the bearing region, and individual sensor placements. The aim was to identify which sensor locations provide the most discriminative information for spindle speed recognition and to determine whether reduced sensor counts could sustain performance. Each configuration was assessed using a consistent cross-alloy validation protocol, allowing examination of sensor location contributions both within a single data distribution and regarding transferability between Al 6061 and Al 7075 material domains. This analysis is important for developing more streamlined and practical vibration monitoring systems.

#### 2.9.3. Top-k Feature Reduction

In the top-k feature reduction analysis, the impact of reducing the number of features on model performance was evaluated. To this end, features were ranked according to model-based importance, and more compact feature sets consisting of the top *k* most important features were created. The ranking was derived from feature importance values obtained from source-trained benchmark models across both transfer directions. Target-domain predictions and performance metrics were not used in the ranking procedure. The Top-k analysis was conducted as a supplementary dimensionality-reduction assessment and was not used to determine the final spindle-related LightGBM configuration. In general, top-k feature selection is expressed as shown in Equation (16):

*X_top_*_-*k*_ = {*x_j_*∣*rank*(*x_j_*) ≤ *k*} (16)

In this context, *X_top_*_-*k*_ refers to the subset of features consisting of the top *k* most important features, and *rank*(*x_j_*) indicates the position of the *j*th feature within the importance ranking. This analysis assessed the extent to which the model depends on a high-dimensional feature space and whether similar performance could be achieved with a reduced feature set. All ablation analyses employed the same cross-validation protocol and performance metrics, ensuring comparability. Consequently, the effects of frequency band selection, sensor configuration, and feature reduction strategies on final model performance were evaluated within a consistent framework. The results demonstrate that the final configuration was determined by algorithm selection, a physically meaningful frequency-domain representation, preservation of sensor location information, and compact feature selection.

### 2.10. Explainability Analysis

An explainability analysis was conducted to interpret the decision-making mechanism of the final model and to identify which autospectrum features support its classification performance. Model-based feature importance and SHAP-based global importance analyses were employed together. The explainability analysis focused exclusively on the configuration that included the selected spindle-related autospectrum descriptors and the LightGBM model. Model-based feature importance analysis assessed which features the LightGBM model utilized most effectively within its decision trees, revealing the relative contributions of each feature to the model’s internal structure. Recognizing that internal importance values alone may be insufficient, SHAP analysis was also performed to evaluate each feature’s contribution to the prediction output in greater detail. SHAP-based global importance analysis quantified the average impact of features on model predictions. This combined approach enabled an assessment of whether the final model’s decisions were based on physically meaningful frequency-domain features, such as harmonic-band, tooth-passing-related, peak-distance, and sensor-specific autospectrum descriptors. By jointly interpreting the feature importance and SHAP results, the evaluation considered both the classification performance and the physical interpretability of the model’s decision-making structure for the spindle speed recognition task.

## 3. Results

This section presents the rationale for selecting cross-alloy spindle speed recognition as the final classification task, based on preliminary target screening results. It then reports benchmark model comparisons, final LightGBM model performance, bidirectional material transfer results, and baseline configuration comparisons. The model’s robustness is evaluated through frequency band, sensor, and top-k feature ablation studies. Finally, the decision-making mechanism of the model, grounded in physically meaningful autospectrum features, is explained using feature importance and SHAP analyses.

### 3.1. Preliminary Target Screening and Final Task Selection

Prior to the spindle speed recognition task, a preliminary cross-alloy scan was performed on Al 6061 and Al 7075 materials using a range of process parameters. The purpose of this scan was to determine the classification target with the greatest stability and highest transferability for subsequent analyses. The results indicated substantial differences in transferability among the process parameters. Preliminary screening indicated that spindle speed provided the most consistent cross-alloy behavior among the evaluated process parameters. Feed rate and depth of cut were therefore retained as auxiliary screening tasks, while spindle speed was selected for the subsequent detailed benchmark and final-model analyses. This spindle speed task was then examined in detail during benchmark and final model analyses. In contrast, feed rate and depth of cut were not designated as primary targets but were used for supplementary and exploratory evaluations. The preliminary scan results for these supplementary tasks are shown in Table 7.

Table 7 indicates that the feed rate demonstrated moderate transferability, whereas the depth of cut task showed limited cross-alloy generalizability. Consequently, subsequent analyses, such as model comparisons, frequency band ablations, sensor ablations, top-k feature reduction analyses, confusion matrix evaluations, and SHAP-based explainability studies, are focused on the spindle speed recognition task.

### 3.2. Benchmark Comparison of Machine Learning Models

Following the identification of spindle speed as the classification target, a comprehensive benchmark analysis was performed to assess the cross-alloy generalization performance of multiple machine learning algorithms. All models were evaluated using an identical all-feature representation to ensure a fair and consistent initial comparison. This methodology facilitated the investigation of whether observed performance differences were attributable to model architecture, decision mechanisms, or classification capabilities. The benchmark included linear models, kernel-based methods, an artificial neural network model, and tree-based ensemble algorithms. Specifically, the models assessed were Logistic Regression, RBF-SGD, Random Forest, ExtraTrees, HistGradientBoosting, MLP, LightGBM, and XGBoost. Comparative evaluation was conducted using metrics such as accuracy, balanced accuracy, macro-F1, MCC, Cohen’s kappa, ROC-AUC, PR-AUC, log-loss, and Brier score. The complete benchmark results are provided in Table 8.

Benchmark results indicate that tree-based boosting and ensemble models generally deliver superior performance. XGBoost achieved the highest Macro-F1 score, Random Forest produced the strongest results in terms of balanced accuracy and MCC, and ExtraTrees yielded the highest ROC-AUC. However, the all-feature representation was not the optimal solution. This finding demonstrates that model success is not determined solely by the use of high-dimensional features. As subsequent analyses will show, spindle-related autospectrum descriptors, which are directly relevant to the task, are more effective.

### 3.3. Final Model Performance

After the benchmark comparison, the final model was evaluated using spindle-related autospectrum descriptors. The selected configuration included the spindle-related frequency band feature set, the LightGBM model, and the default threshold of 0.50. The model exhibited strong and balanced performance in the cross-alloy spindle-speed recognition task, achieving a Macro-F1 score of 0.8701, balanced accuracy of 0.8708, MCC of 0.7500, and ROC-AUC of 0.9615. Additionally, the PR-AUC (0.9631), log-loss (0.2938), and Brier score (0.0921) indicate high probabilistic discrimination capability in addition to robust classification performance. These findings suggest that using spindle-related frequency-domain features, which are directly relevant to the task, improves model performance compared to an all-feature representation. The effectiveness of the final model is attributed to both the algorithm and the selection of physically meaningful autospectrum features. The configuration, determined through frequency-band ablation analysis, was evaluated as the final model with a decision threshold of 0.50. The corresponding performance metrics are presented in Table 9.

**Figure 5:**
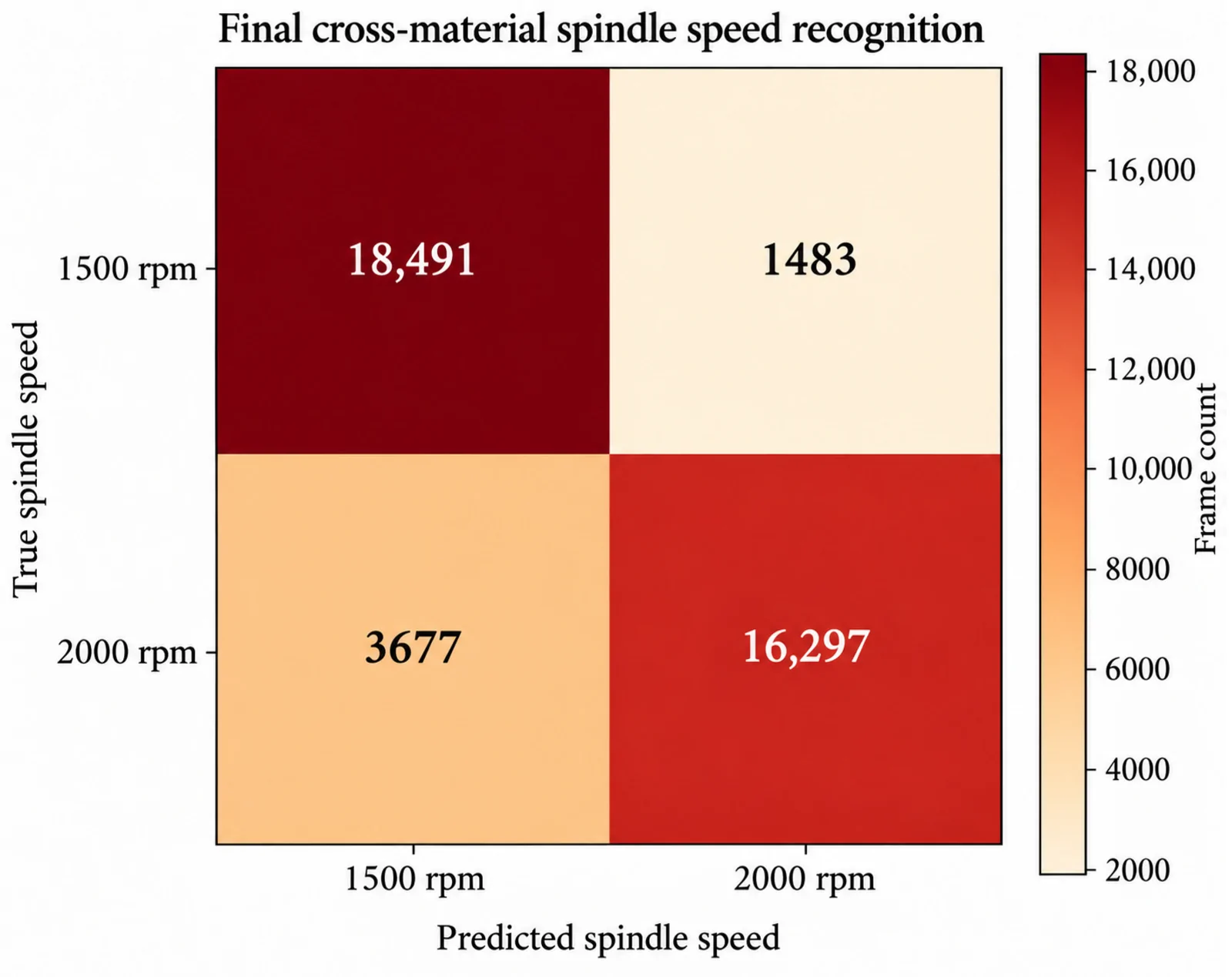
Final pooled confusion matrix for the selected LightGBM model.

Table 9 shows that the final LightGBM model achieves both balanced and robust classification performance when spindle-related autospectrum descriptors are utilized. The similarity between the Macro-F1 and balanced accuracy values suggests that the model does not exhibit significant bias toward either spindle-speed class. In comparison to the all-feature LightGBM benchmark result in Table 8, the observed performance improvement is attributed not only to model selection but also to the use of a physically meaningful set of spindle-related features identified through frequency-band ablation. To further assess the class-specific error distribution of the final model, the pooled confusion matrix for the selected LightGBM model is provided in Figure 5.

Figure 5 presents the frame-level classification performance of the final model, which was developed using spindle-related autospectrum descriptors, across two spindle-speed classes. The results show that the model reliably distinguishes between the 1500 rpm and 2000 rpm classes. The confusion matrix is interpreted here as a quantitative measure of whether the two controlled spindle states remain separable after alloy-domain transfer, rather than as evidence for recovering an unknown spindle-speed command. To avoid evaluating the final model based solely on a single performance metric, subsequent sections offer a comprehensive assessment, including bidirectional material transfer, baseline configuration comparisons, frequency band ablation, sensor ablation, top-k feature reduction, and SHAP-based explainability analyses. This approach ensures that the evaluation considers not only the accuracy of the final configuration but also its generalizability, physical interpretability, and consistency in reduced-dimensional or sensor-limited scenarios.

### 3.4. Bidirectional Cross-Alloy Transfer Performance

To determine whether the final model demonstrated success exclusively in a single direction of material transfer, the selected configuration, which included spindle-related autospectrum descriptors and LightGBM, was assessed using a bidirectional cross-alloy validation approach. Specifically, the model was first evaluated for the Al 6061 → Al 7075 transfer scenario, followed by the Al 7075 → Al 6061 scenario. The results of the bidirectional transfer are provided in Table 10.

**Figure 6:**
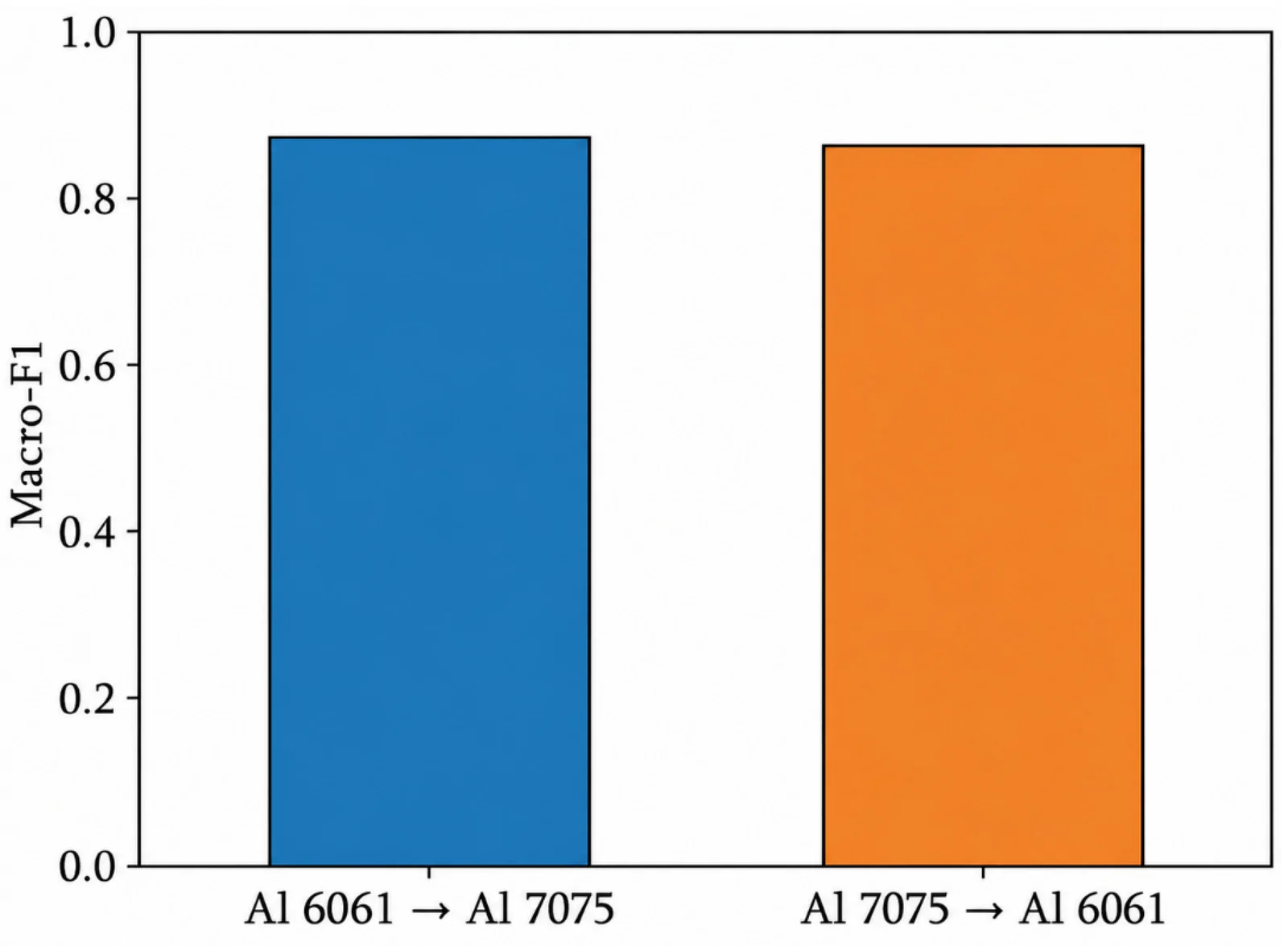
Macro-F1 comparison for the two cross-alloy transfer directions.

Table 10 demonstrates that the final model achieved balanced and comparable performance in both transfer directions. This result indicates that comparable performance was obtained in both examined transfer directions. Accordingly, spindle-related autospectrum features provide a transferable representation between Al 6061 and Al 7075 under the investigated conditions. The observation that Macro-F1, balanced accuracy, and MCC values remained consistent in both directions supports the conclusion that the final configuration maintains stable classification behavior during cross-alloy transfer. Figure 6 is included to visually compare the consistency of the final model across the two opposite transfer directions.

Figure 6 compares the Macro-F1 performance of the selected LightGBM model for the two transfer directions, Al 6061 → Al 7075 and Al 7075 → Al 6061. The close agreement between these two values indicates that reversing the source and target alloys does not substantially change the classification performance in the spindle-speed recognition task. These findings indicate consistent model performance within the investigated bidirectional cross-alloy setting. Therefore, spindle-related autospectrum descriptors provide a stable and transferable feature representation between Al 6061 and Al 7075 under the examined experimental conditions.

### 3.5. Key Configuration Comparison

To clarify the rationale for selecting the “spindle-related bands + LightGBM” combination as the final model, four representative configurations were evaluated: all features + XGBoost, spindle-related bands + LightGBM, top-15 features + LightGBM, and right-bed-only + ExtraTrees. This comparison demonstrates that the observed performance improvement results from both the choice of model and the selection of physically meaningful frequency-domain features. The results of this configuration comparison are provided in Table 11.

**Figure 7:**
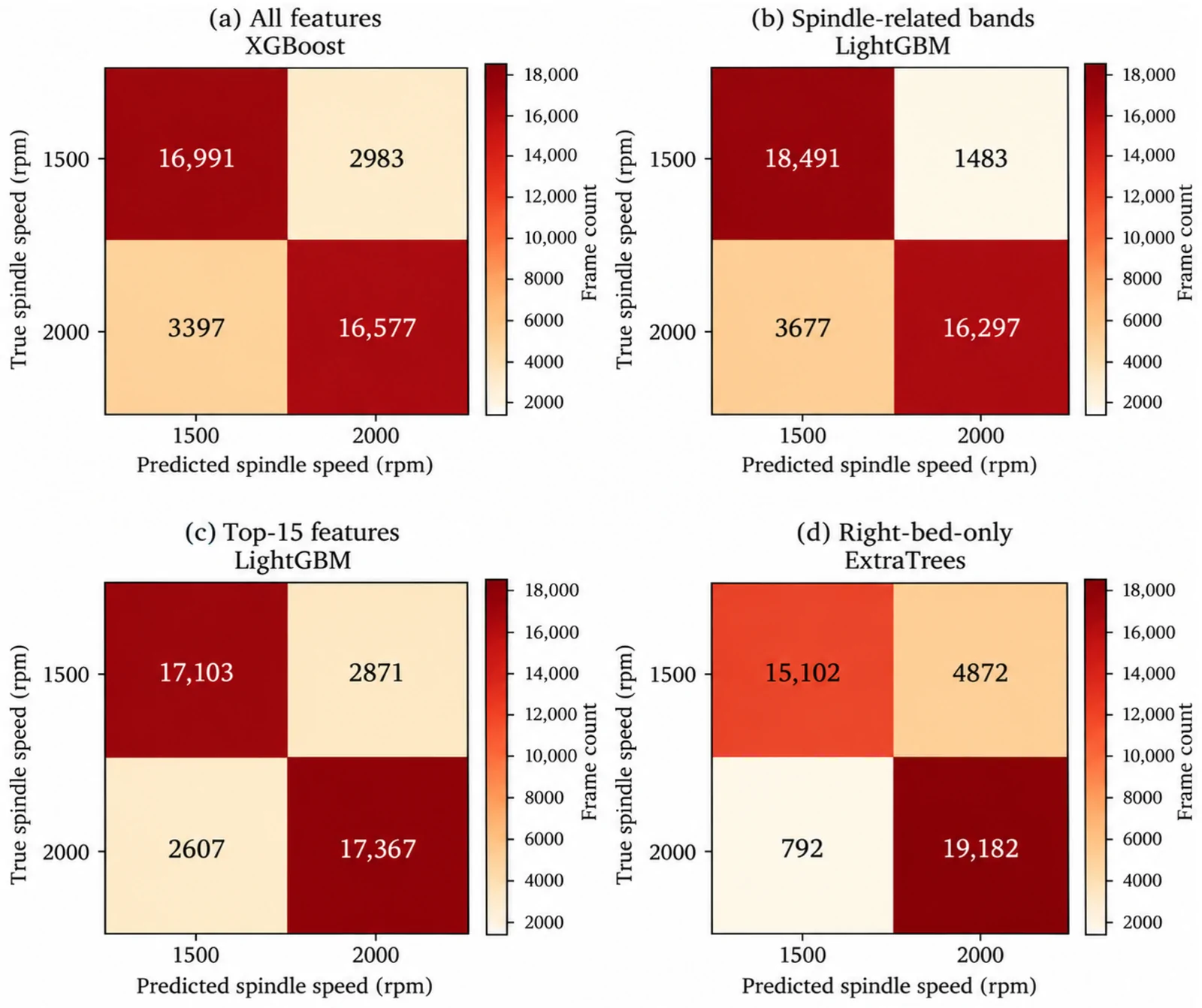
Key configuration confusion matrix panel.

Table 11 demonstrates that the spindle-related bands combined with the LightGBM configuration achieved the highest performance. While the XGBoost model using the all-feature representation provided a strong benchmark, the physically selected spindle-related autospectrum descriptors outperformed it. This finding suggests that aggregating all features does not necessarily yield the most effective representation; instead, focusing on spectral regions directly relevant to the task enhances performance. The top-15 features with LightGBM also achieved results comparable to the final model, indicating that model success does not require a high-dimensional feature space and that robust outcomes are attainable with more compact feature sets. Additionally, the right-bed-only with ExtraTrees configuration demonstrated that meaningful classification performance can be sustained even with a reduced-sensor setup. When evaluated using pooled target-alloy predictions, the right-bed-only configuration achieved Macro-F1 values of 0.8673 for Al 6061 → Al 7075 and 0.8460 for Al 7075 → Al 6061. Across the paired cutting-condition groups, Macro-F1 ranged from 0.7936 to 0.9419 and from 0.7141 to 0.9629, respectively. These results indicate comparable overall transfer performance in both directions, although greater variability was observed across individual cutting conditions. Figure 7 presents a comparison of the confusion matrices for these four key configurations.

Figure 7 presents the class-based error distributions for the all-feature benchmark, the final spindle-related feature set, the top-15 feature reduction, and the right-bed-only sensor configuration. The results indicate that the final model achieves balanced performance across overall metrics and effectively distinguishes between the 1500 rpm and 2000 rpm classes. These findings motivate a detailed examination in the subsequent section of the effects of frequency band selection, sensor configuration, and feature dimensionality reduction on final performance. This comparison demonstrates that the final configuration depends not only on model selection but also on the incorporation of physically meaningful spindle-related autospectrum descriptors.

### 3.6. Frequency-Band, Sensor, and Top-k Feature Ablation

**Figure 8:**
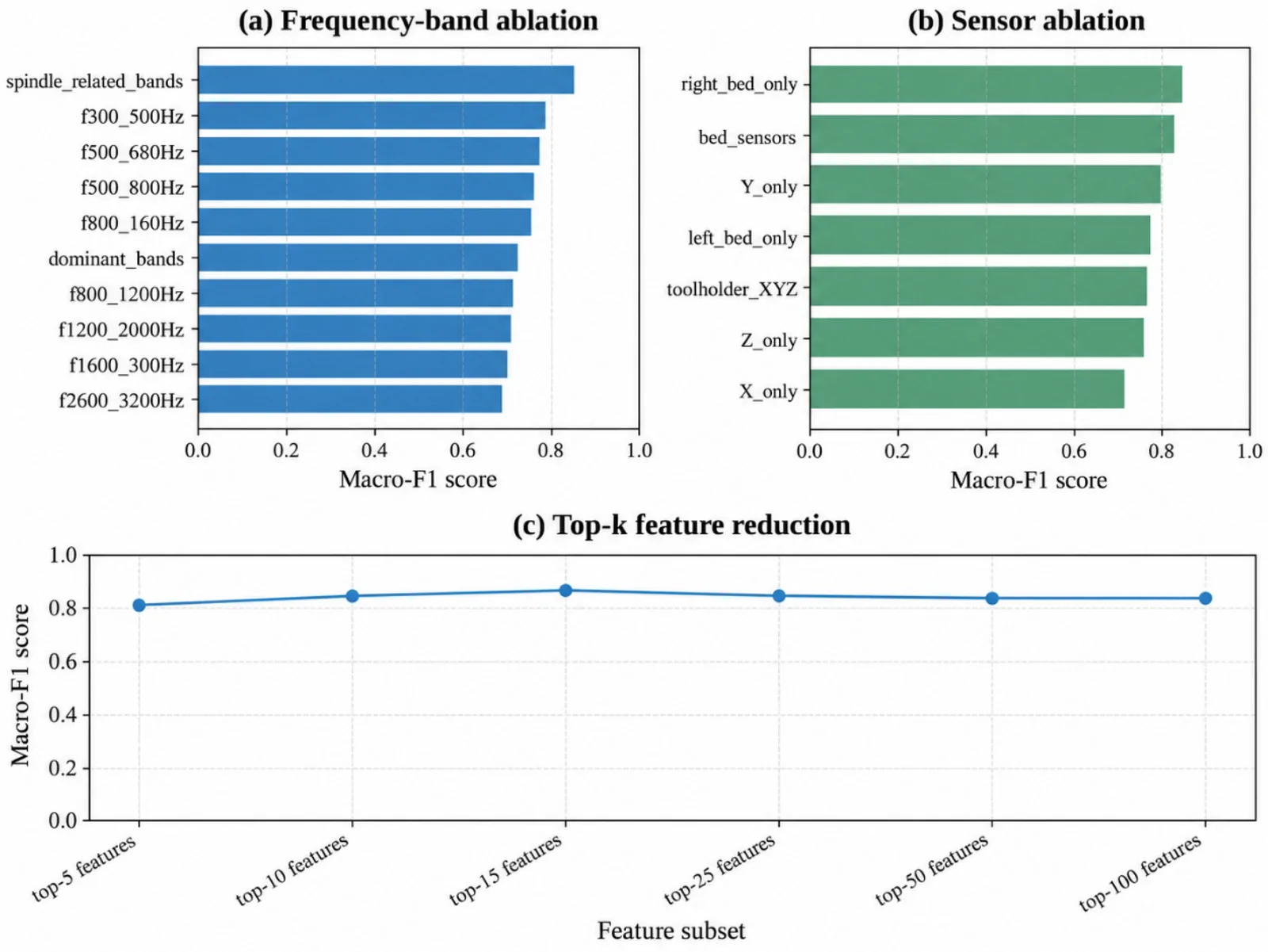
Frequency-band, sensor, and top-k feature ablation comparison.

The performance of the final configuration was evaluated in detail by examining the effects of frequency band selection, sensor configuration, and feature dimensionality reduction through ablation analyses. These analyses aimed to determine whether model performance depended exclusively on the entire high-dimensional feature pool or could be sustained using more compact and physically meaningful feature sets relevant to the task. The ablation results are shown in Figure 8.

Figure 8 indicates that the highest performance was obtained using the “spindle-related bands” feature set. This finding suggests that employing all available features does not necessarily provide the most effective representation; instead, selecting frequency regions with physical relevance is more advantageous for spindle-speed recognition. Additionally, compact feature sets identified through “top-k” feature reduction analysis achieved performance levels comparable to the final model, indicating that the model does not rely on a high-dimensional feature space. Sensor ablation experiments further demonstrate that competitive results are attainable with reduced sensor configurations. Collectively, these results indicate that the final model’s performance is influenced not only by the choice of algorithm but also by strategies for frequency band selection, sensor configuration, and feature dimensionality reduction. Therefore, spindle-related autospectrum descriptors represent the most effective and physically interpretable feature set. The final spindle-related feature set reduced the input dimensionality from 566 to 170 features, corresponding to a 70.0% reduction. This substantial dimensionality reduction decreases the amount of feature information processed by the classifier and consequently reduces the computational and memory requirements of the proposed model.

### 3.7. Feature Importance and SHAP-Based Explainability

Feature importance and SHAP analyses were jointly evaluated to interpret the decision-making mechanism of the final model. These analyses aimed to assess not only the model’s performance but also whether its classification decisions relied on physically meaningful autospectrum features. The most significant features identified by the final LightGBM model are listed in Table 12.

As shown in Table 12, the most influential features of the final model are predominantly derived from spindle-related spectral descriptors. This indicates that the model utilizes task-related frequency-domain components during its decision-making process. The feature importance results and the SHAP global importance plot are presented in Figure 5.

**Figure 9:**
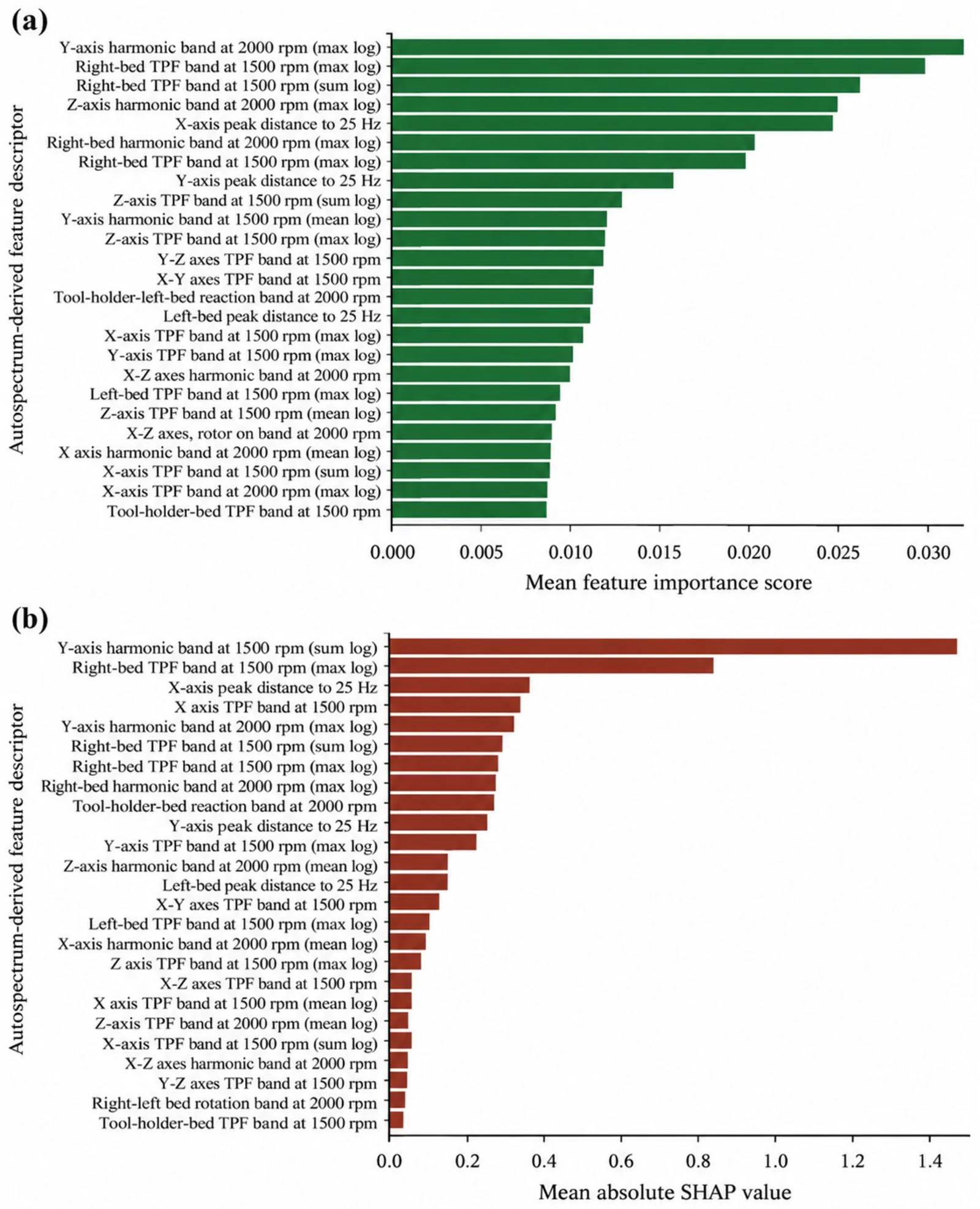
Explainability analyses for the final LightGBM model. (**a**) Model-based feature importance results for the final LightGBM model. (**b**) SHAP global importance results for the final LightGBM model.

Figure 9 illustrates that features such as the harmonic band, tooth-passing-related band, peak distance, and spindle-related autospectrum (which depends on sensor location) are prominent in the decision-making process of the final LightGBM model. The alignment between model-based feature importance and SHAP global importance results suggests that the model’s classification decisions rely on physically meaningful frequency-domain representations. The high ranking of features from the *Y*-axis, *X*-axis, and right-bearing sensors indicates that vibration data from both the tool zone and the bearing zone contribute complementary information to the spindle-speed recognition task. These results support the conclusion that the final configuration achieves both high performance and an interpretable decision-making mechanism. The prominence of these SHAP-ranked features is physically consistent with the spindle-speed-dependent rotational, tooth-passing, and harmonic frequencies defined in Section 2.9.1. To further assess the stability of the SHAP-based interpretation, feature rankings were examined separately across the two transfer directions and after stratifying the SHAP samples by the true spindle-speed class.

As shown in Table 13, the overall SHAP feature-ranking structure was substantially consistent between the two transfer directions and highly consistent when the SHAP samples were stratified by the true spindle-speed class. Some transfer-direction-specific differences were nevertheless observed in the highest-ranked individual features.

## 4. Discussion

The central question addressed in this study is whether physically anchored spindle-synchronous vibration signatures remain discriminative when the workpiece alloy changes. Spindle speed was therefore used as a controlled process-state label rather than as an unknown variable requiring estimation. The classification framework provided a quantitative means of evaluating this transferability under bidirectional Al 6061 ↔ Al 7075 domain shifts. Accordingly, the principal finding is not simply that 1500 and 2000 rpm can be distinguished from their characteristic spectral components, but that spindle-synchronous vibration representations retain substantial discriminative information after the alloy domain is changed. Previous research on vibration-based process monitoring typically relies on train-test splits or random data partitioning within a single material domain. Although these methods are effective for evaluating model performance, they provide only limited insight into generalization across material domains. The primary contribution of this study is the evaluation of the model both within the same data distribution and across bidirectional cross-alloy transfer scenarios, specifically from Al 6061 to Al 7075 and vice versa. This approach establishes a more rigorous and realistic generalization scenario for spindle speed recognition. The use of Al 6061 and Al 7075 in this study should therefore be interpreted as a controlled material-domain change rather than as an attempt to characterize the intrinsic material properties of the two alloys. The autospectrum observations showed material- and condition-dependent differences in the overall spectral energy distribution, whereas the cross-alloy results demonstrated that spindle-related rotational, tooth-passing, and harmonic descriptors retained discriminative information across the two alloy domains. Thus, the material-related insight provided by the present study concerns the transferability of vibration-based machining signatures under an alloy change, rather than a direct investigation of alloy properties or machinability. Establishing relationships between specific mechanical or thermophysical material properties and the corresponding vibration response remains an important direction for future work. Benchmark analyses compared several machine learning models using an identical all-feature representation. Tree-based models, including XGBoost, Random Forest, and ExtraTrees, demonstrated strong performance at this stage. However, the optimal result was achieved not with the all-feature approach, but by employing spindle-related autospectrum descriptors selected through frequency-band ablation. The final LightGBM model attained a Macro-F1 score of 0.8701, balanced accuracy of 0.8708, MCC of 0.7500, and ROC-AUC of 0.9615. These results indicate that model performance is influenced by both algorithm selection and the use of frequency-domain features that are physically relevant to the task. Bidirectional cross-alloy transfer results further support this conclusion, as the final model achieved comparable performance metrics for both transfer directions. The consistently high and balanced Macro-F1, balanced accuracy, MCC, ROC-AUC, and PR-AUC values in both directions indicate that the model did not simply learn patterns specific to a single material transfer direction. This finding supports the conclusion that spindle-related autospectrum descriptors provide a consistent and transferable representation between the two examined aluminum alloys under the investigated conditions. Baseline configuration comparisons further clarified the rationale for the final approach: the spindle-related bands combined with LightGBM achieved the highest overall performance relative to the all features with XGBoost, top-15 features with LightGBM, and right-bed-only with ExtraTrees configurations. Notably, the top-15 features configuration performed nearly as well as the final model, suggesting that the model’s effectiveness does not depend solely on a high-dimensional feature set. These results demonstrate that compact and interpretable feature sets can provide sufficient representational power for spindle speed recognition. Notably, the competitive performance of the right-bed-only sensor configuration suggests that vibration data from the bed region effectively capture process dynamics associated with spindle speed through structural transmission paths. As a result, a multi-sensor setup may not be necessary in all cases, which benefits the development of low-cost, less complex, and industrially viable monitoring systems. Ablation analyses indicate that final performance is not solely due to a high number of features. When considering frequency bands, sensor configurations, and top-k feature reduction collectively, spindle-related autospectrum descriptors emerge as the most effective and interpretable feature representations. This suggests that the model leverages task-relevant physical frequency-domain components rather than a generic, high-dimensional feature pool. Explainability analyses further show that the model’s decision-making is grounded in physically meaningful autospectrum features. The prominence of features such as harmonic bands, tooth-passing-related bands, peak distances, and sensor-location-dependent spindle-related characteristics, as evidenced by feature importance and SHAP results, supports the conclusion that the model bases its decisions on frequency-domain components linked to process physics rather than on random or purely statistically discriminative patterns. This highlights that the proposed approach provides an interpretable machine learning framework, not one focused solely on performance. However, the study has certain limitations. First, the evaluation is limited to two aluminum alloys, so the results should be interpreted as a cross-alloy spindle speed recognition framework validated under specific experimental conditions, rather than as a generalized CNC milling model. Therefore, the reported generalization is limited to transfer between Al 6061 and Al 7075 under the investigated cutting conditions of 1500–2000 rpm spindle speed, 0.05–0.10 mm/rev feed rate, and 0.5–1.0 mm depth of cut. Generalization to other material systems, wider cutting-parameter ranges, tool geometries, and machine configurations requires additional experimental validation. Second, analyses were conducted at the frame level, and these frames were not treated as independent machining experiments; thus, findings should be considered within a cross-alloy and condition-aware evaluation framework. Third, because the study relied on autospectrum representations, time-domain signal dynamics and phase information were not directly assessed. Future work should include broader validation across diverse materials, tool geometries, tool wear states, cutting parameters, and machine tool conditions. Overall, the results indicate that the physics-informed autospectrum feature selection approach provides balanced and interpretable performance within the investigated cross-alloy spindle speed recognition setting. Consistency across benchmark, bidirectional transfer, key configuration comparison, ablation, and SHAP analyses demonstrates that the final configuration is supported by generalizability, compactness, applicability, and explainability, rather than by a single performance metric.

## 5. Conclusions

Preliminary screening identified spindle speed as the optimal classification objective, demonstrating superior and more stable cross-alloy transferability compared to feed rate and depth of cut. The final model configuration employed LightGBM, trained on spindle-related autospectrum descriptors selected through frequency-band ablation. This model achieved balanced and consistent classification performance within the investigated cross-alloy setting, with Macro-F1 of 0.8701, balanced accuracy of 0.8708, MCC of 0.7500, and ROC-AUC of 0.9615. Bidirectional transfer analyses confirmed consistent model performance in both Al 6061 to Al 7075 and Al 7075 to Al 6061 directions. Ablation studies indicated that success was not due to high-dimensional feature use but rather to the selection of physically meaningful, spindle-related frequency-domain features. Top-k feature reduction demonstrated that near-optimal performance could be maintained with compact feature sets. Sensor ablation further showed that competitive results were achievable with reduced sensor configurations. Feature importance and SHAP analyses revealed that the model’s decision process relied on spindle-related autospectrum features, including harmonics, tooth-passing frequencies, peak distances, and sensor locations. These findings support the conclusion that the proposed model offers a physically interpretable decision structure. Overall, the physics-informed autospectrum feature selection approach provides a balanced and interpretable framework for cross-alloy spindle speed recognition within the investigated experimental scope. The results are limited to the current experimental scope, and future research should validate the approach across diverse materials, tool wear conditions, cutting regimes, machine tools, and real-time monitoring scenarios.

## Figures and Tables

**Table 1:** Technical properties of the CNC milling machine used in the experiments.

Category	Parameter	Value
**Travels**	X-Axis travel	762 mm
	Y-Axis travel	406 mm
	Z-Axis travel	406 mm
**Spindle Region**	Max spindle speed	6000 rpm
	Max spindle power	5.6 kW
	Max torque	1200 rpm
**Table Region**	Table length	1213 mm
	Table width	267 mm
	Max table load	159 kg
**Feed rates**	Max cutting feed rate	10.2 m/min
**Axis Motors**	Max thrust force	8896 N
**Tool Changer**	Max tool weight	5.4 kg
	Max tool diameter	89 mm

**Table 2:** Cutting conditions used in the CNC milling experiments.

Condition ID	Feed Rate (mm/rev)	Spindle Speed (rpm)	Cutting Speed (m/min)	Depth of Cut (mm)
D1	0.05	1500	75.4	0.5
D2	0.05	1500		1.0
D3	0.05	2000	100.5	0.5
D4	0.05	2000		1.0
D5	0.10	1500	75.4	0.5
D6	0.10	1500		1.0
D7	0.10	2000	100.5	0.5
D8	0.10	2000		1.0

**Table 3:** Vibration data acquisition and autospectrum data structure.

Item	Description
Measurement system	Brüel & Kjær 3560-B IDA with PULSE environment
Data representation	Processed autospectrum frames
Signal domain	Frequency domain
Experimental files	16
Vibration channels	5
Tool-holder/spindle channels	X, Y, Z
Bed-region channels	Left bed, right bed
Frequency range	0–3.2 kHz
Frequency resolution	4 Hz
Frequency bins per frame	801
Frame-level observations	39,948
Total autospectrum values	159,991,740
Main analysis unit	Frame-level autospectrum observation

**Table 4:** Label structure used for frame-level autospectrum observations.

Label Field	Values	Role in the Study
**Material**	Al 6061, Al 7075	Cross-alloy domain variable
**Condition ID**	D1–D8	Cutting-condition identifier
**Feed rate**	0.05, 0.10 mm/rev	Auxiliary screening target/condition descriptor
**Spindle speed**	1500, 2000 rpm	Main classification target
**Depth of cut**	0.5, 1.0 mm	Auxiliary screening target/condition descriptor
**Vibration channel**	X, Y, Z, left bed, right bed	Sensor-location descriptor
**Frame index**	Frame-level order within each file	Observation identifier

**Table 5:** Machine learning models evaluated in this study.

Model	Model Family	Role in the Study
Logistic Regression	Linear model	Baseline linear classifier
RBF-SGD	Kernel-based linear approximation	Nonlinear baseline classifier
Random Forest	Bagging ensemble	Tree-based ensemble benchmark
ExtraTrees	Randomized tree ensemble	Tree-based ensemble benchmark
HistGradientBoosting	Gradient boosting	Boosting-based benchmark
MLP	Artificial neural network	Neural network baseline
LightGBM	Gradient boosting	Candidate final model
XGBoost	Gradient boosting	Strong boosting benchmark

**Table 6:** Main hyperparameter settings of the machine learning models.

Model	Main Settings and Notes
Logistic Regression	C = 1.0; LBFGS solver; 4000 iterations; Balanced class weights
RBF-SGD	RBF mapping with γ = 1/*p*; 1200 components; log-loss SGD; α = 10^−4^; 2500 iterations; Balanced class weights
ExtraTrees	700 trees; square-root feature sampling; minimum leaf size = 2; Balanced class weights
Random Forest	500 trees; square-root feature sampling; minimum leaf size = 2; Balanced class weights
HistGradientBoosting	600 boosting iterations; learning rate = 0.035; 31 leaf nodes; L_2_ = 0.01; Fixed boosting setting
MLP	Hidden layers: 256–128–64; ReLU activation; α = 10^−4^; learning rate = 5 × 10^−4^; 300 iterations; Early stopping enabled
LightGBM	900 trees; learning rate = 0.025; 31 leaves; row/column sampling = 0.90; L_1_ = 0.05, L_2_ = 0.05; Balanced class weights
XGBoost	800 trees; depth = 4; learning rate = 0.025; row/column sampling = 0.90; L_1_ = 0.03, L_2_ = 1.0; Binary logistic objective

**Table 7:** Preliminary screening results for auxiliary process-parameter recognition tasks.

Target Parameter	Best Model	Threshold Policy	Macro-F1	Balanced Accuracy	Final Role in the Study
Feed rate	ExtraTrees	source_val_f1	0.6526	0.6566	Exploratory/supplementary
Depth of cut	ExtraTrees_regularized	default_0p50	0.5929	0.6009	

**Table 8:** Benchmark performance of machine learning models for cross-alloy spindle speed recognition using all-feature representation.

Rank	Model	Model Family	Macro-F1	Balanced Accuracy	MCC	ROC-AUC
1	XGBoost	Gradient boosting	0.8391	0.8403	0.6880	0.9275
2	Random Forest	Bagging ensemble	0.8368	0.8426	0.7090	0.9347
3	ExtraTrees	Randomized ensemble	0.8340	0.8389	0.6999	0.9370
4	LightGBM	Gradient boosting	0.8275	0.8296	0.6725	0.9332
5	HistGradientBoosting	Gradient boosting	0.8259	0.8272	0.6644	0.9297
6	RBF-SGD	Kernel-based linear model	0.8180	0.8243	0.6734	0.9234
7	MLP	Artificial neural network	0.8124	0.8165	0.6552	0.9299
8	Logistic Regression	Linear model	0.8081	0.8113	0.6353	0.8978

**Table 9:** Final model performance obtained using spindle-related autospectrum descriptors.

Metric	Value
Accuracy	0.8708
Balanced accuracy	0.8708
Macro-F1	0.8701
MCC	0.7500
Cohen’s κ	0.7417
ROC-AUC	0.9615
PR-AUC	0.9631
Log-loss	0.2938
Brier score	0.0921

**Table 10:** Bidirectional transfer performance of the final model.

Metric	Al 6061 → Al 7075	Al 7075 → Al 6061
No. of frames	19,972	19,976
Accuracy	0.8753	0.8664
Balanced accuracy	0.8753	0.8664
Macro-F1	0.8748	0.8661
MCC	0.7566	0.7360
Cohen’s κ	0.7506	0.7328
ROC-AUC	0.9623	0.9590
PR-AUC	0.9635	0.9614

**Table 11:** Key configuration comparison for cross-alloy spindle-speed recognition.

Configuration	Model	Macro-F1	Balanced Accuracy	MCC	ROC-AUC
Spindle-related bands	LightGBM	0.8701	0.8708	0.7500	0.9615
Top-15 features	LightGBM	0.8624	0.8629	0.7317	0.9448
Right-bed-only	ExtraTrees	0.8528	0.8582	0.7460	0.9527
All features	XGBoost	0.8391	0.8403	0.6880	0.9275

**Table 12:** The most influential autospectrum features in the final LightGBM model.

Rank	Feature Descriptor	Importance	Mean |SHAP|	SHAP Rank
1	Y 2000 rpm harmonic band max log	0.0319	0.3542	5
2	Right bed TPF 1500 rpm band ratio	0.0300	0.3265	7
3	Right bed TPF 1500 rpm band sum log	0.0265	0.3231	8
4	Z 2000 rpm harmonic band max log	0.0251	0.1674	16
5	X distance to 25 Hz	0.0251	0.3863	3
6	Right bed 2000 rpm harmonic band max log	0.0208	0.3173	9
7	Right bed TPF 1500 rpm band max log	0.0203	0.8971	2
8	Y distance to 25 Hz	0.0155	0.3029	11
9	Z TPF 1500 rpm band sum log	0.0126	0.0835	30
10	Y 1500 rpm harmonic band ratio	0.0115	1.5241	1

**Table 13:** SHAP feature-ranking stability across transfer directions and spindle-speed classes.

Comparison	Spearman ρ	Top-10 Overlap (%)
Al 6061 → Al 7075 vs. Al 7075 → Al 6061	0.7158	50
1500 rpm vs. 2000 rpm	0.9755	60

## Data Availability

The data supporting the findings of this study were obtained experimentally and are available from the author upon reasonable request.
